# TLR4 activation induces IL-1β release via an IPAF dependent but caspase 1/11/8 independent pathway in the lung

**DOI:** 10.1186/s12931-014-0087-0

**Published:** 2014-08-02

**Authors:** Suffwan Eltom, Maria G Belvisi, Liang Yew-Booth, Bilel Dekkak, Sarah A Maher, Eric D Dubuis, Victoria Jones, Kate A Fitzgerald, Mark A Birrell

**Affiliations:** 1 Respiratory Pharmacology, National Heart and Lung Institute, Faculty of Medicine, Imperial College London, Exhibition Road, London SW7 2AZ, UK; 2 University of Massachusetts, Division of Infectious Diseases & Immunology, Worcester 01605, MA, USA

**Keywords:** TLR4, IPAF, ASC, IL-1 release

## Abstract

**Background:**

The IL-1 family of cytokines is known to play an important role in inflammation therefore understanding the mechanism by which they are produced is paramount. Despite the recent plethora of publications dedicated to the study of these cytokines, the mechanism by which they are produced in the airway following endotoxin, Lipopolysaccharide (LPS), exposure is currently unclear. The aim was to determine the mechanism by which the IL-1 cytokines are produced after LPS inhaled challenge.

**Methods:**

Mice were challenged with aerosolised LPS, and lung tissue and bronchiolar lavage fluid (BALF) collected. Targets were measured at the mRNA and protein level; caspase activity was determined using specific assays.

**Results:**

BALF IL-1b/IL-18, but not IL-1a, was dependent on Ice Protease-Activating Factor (IPAF), and to a lesser extent Apoptosis-associated Speck-like protein containing a CARD (ASC). Interestingly, although we measured an increase in mRNA expression for caspase 1 and 11, we could not detect an increase in lung enzyme activity or a role for them in IL-1a/b production. Further investigations showed that whilst we could detect an increase in caspase 8 activity at later points in the time course (during resolution of inflammation), it appeared to play no role in the production of IL-1 cytokines in this model system.

**Conclusions:**

TLR4 activation increases levels of BALF IL-1b/IL-18 via an IPAF dependent and caspase 1/11/8 independent pathway. Furthermore, it would appear that the presence of IL-1a in the BALF is independent of these pathways. This novel data sheds light on innate signalling pathways in the lung that control the production of these key inflammatory cytokines.

## Background

The IL-1 family of cytokines are known to possess potent pro-inflammatory properties [1],[2] and controlling their production and release is vital to maintaining a healthy lung [3],[4]. The family contains IL-1α and IL-1β, which both activate the same receptor, IL-1R, [5] and IL-18 which activates the IL-18R [6],[7]. All three cytokines are transcribed into pro-forms and, whereas pro-IL-1α is biologically active, pro-IL-1β and pro-IL-18 require cleaving into their mature forms [8]–[11]. The production of mature, active forms of IL-1β and IL-18 in many scenarios is thought to involve a *2 step process*: firstly the transcription and translation of the pro-forms via a stimulus such as a TLR agonist (i.e. LPS on TLR4) and then a second stimulus that triggers the cleavage and subsequent release of the mature form (i.e. ATP activating P2X_7_ receptors) [12]–[16]. Pro-IL-1β and pro-IL-18, and not Pro-IL-1α, can be cleaved into mature forms by caspase 1 [17]. Mature caspase 1 itself is activated/cleaved from the inactive pro-form [17]. This could be auto-processing [18] or via the actions of other kinases such as caspase 4 (or its murine homolog, caspase 11) [19]. Caspase-11 does not process pro-IL-1β directly [20].

Depending on the stimulus and the conditions, caspase 1 can be recruited into a protein complex known as the inflammasome which can include NALP3 (or NLRP3, PYPAF1, CIAS1), AIM2, IPAF (or NLRC4, TMS1) and ASC [17],[21],[22]. ASC is an adaptor molecule that is required in some instances to achieve partial or total activity of the inflammasome [23]. The key proteins central to IL-1β/IL-18 maturation depends on the stimulus and conditions [24]–[26]. IL-1β and IL-18 have also been shown to be released via caspase 1 independent mechanisms [27]–[29]. Recently evidence has been published to suggest caspase 8 can be involved in IL-1β maturation [30]. Furthermore, in some circumstances it is thought that the pro-forms are released and then cleaved into the mature form by a range of enzymes including fungal pathogens [31]; neutrophil proteinase-3 (PR3) [32],[33]; mast cell chymase [34]; matrix metalloproteinases [35]; Cathepsin G [36] and a range of proteases including granzyme A elastase [10],[37],[38].

Despite the recent plethora of publications dedicated to the study of the IL-1 family of cytokines, the mechanism by which they are produced in the airway after endotoxin (LPS) exposure is currently unclear. Typically experiments which investigate this signalling pathway and the production of IL-1β family cytokines involve the administration of lethal doses of LPS and the production of systemic cytokines. However, it could be argued that more moderate levels of LPS exposure, not resulting in lethality, are more clinically relevant. Indeed, these more moderate experimental protocols are known to result in measurable levels of IL-1β in the mouse, rat and human airway [39]–[43]. The aim of this study was to determine the mechanism by which the IL-1 family members are produced following after inhalation of the bacterial mimetic LPS in a murine model.

## Materials and methods

### Mice

All *in vivo* protocols were approved by Imperial College London ethical review process committee and we strictly adhered to the Animals (Scientific Procedures) Act 1986 UK Home Office guidelines. Experiments were performed under a Home office project licence (PPL 70/7212). Male C57bl/6 mice (18-24 g) were originally obtained from Harlan UK Limited (Bicester, UK) and bred in-house; food and water supplied *ad libitum*. Genetically modified mice (knockout, KOs) were back crossed at least 8 times and bred alongside the wild type mice: TLR4 −/−, Myd 88 −/−, P2X_7_ −/−, ASC −/−, NALP3 −/−, IPAF −/− IL-1β −/−, IL-18 −/−, caspase 1 −/− and caspase 11 −/−. Recently it has been established that due to the way they were originally engineered, the caspase 1 KO mice are also deficient in caspase 11 [44]. The KO mice were donated from various laboratories: P2X_7_ from Professor Jean Kanellopoulos from University Paris-Sud; Myd 88 −/−, TLR4 −/− and caspase 1 −/− from the Swiss Immunological Mouse Repository (SwImMR); IL-1b −/− from Professor Yoichiro Iwakura from the University of Tokyo, IL-18 −/− were from Jackson labs, USA; ASC −/−, NALP3 −/−, and IPAF −/− from Professor Kate Fitzgerald (via Professor Clare Bryant, Cambridge University), University of Massachusetts Medical School and caspase 11 −/− from Professor Dixit, Genentech, USA.

### Model system

Mice were challenged with aerosolised saline or sub-maximal LPS (1 mg/ml, *Escherichia coli* serotype 0111:B4 from Sigma, UK [39]) in a perspex box for 30 minutes.

#### Time course study

Wild type mice were culled with an overdose of pentobarbitone (Merial, France, 200 mg/kg, i.p.) at 2, 6, 24, 72 96 and 168 hours after the end of the challenge. Tail tips were harvested and kept at -20°C for possible future genotype confirmation. The trachea was cannulated (Teflon precision dispensing tips, From Adhesive dispensers Ltd, UK) and then lavaged with 0.3 ml RPMI 1640 (Invitrogen, UK) three times, and the lavage fluid pooled. The chest was opened and the lung tissue removed, cleaned and flash frozen in liquid nitrogen. The lavage fluid was centrifuged at 900 g at 4°C for 10 minutes and the supernatant retained for cytokine measurement. The cytokines were measured either by Meso Scale Discovery (MSD, USA) technology or using specific ELISAs from R&D systems, UK.

Gene expression levels were measured according to a method we have previously described (McCluskie et al., 2004). Briefly, RNA was extracted with TRI reagent (Sigma, UK) and samples were reverse transcribed using a master mix (Applied Biosystems, UK) in a PerkinElmer 480 thermal cycler (PerkinElmer Life and Analytical Sciences, USA). Transcriptional expression of target mRNA transcripts in RNA samples were detected by TaqMan real-time quantitative polymerase chain reaction (PCR) with the ABI PRISM 7000 Sequence Detection System (Applied Biosystems, UK). Fluorescent-labelled TaqMan probes for target genes were purchased from Applied Biosystems (UK). Reactions were internally controlled with the 18S rRNA internal control (Applied Biosystems, UK) and performed as multiplex reactions. Validations were performed to ensure the reactions were efficient. Caspase 1 and 8 activity in the lung tissue was measured using specific commercially available assays. The caspase 1 assay and data is presented by Eltom *et al.*[39]. For caspase 8 activity, the same lung tissue cytosolic fractions were assessed using a Caspase-Glo® 8 Assay (Promega, UK) according to the manufacturer’s instructions.

#### Investigative studies

Wild type or GM mice were challenged with vehicle or LPS (as above) and cytokine profile determined. In some cases, target mRNA expression levels and caspase activity was assessed.

## Results

### Time course for LPS driven response: IL-1 family and associated machinery

**Figure 1:**
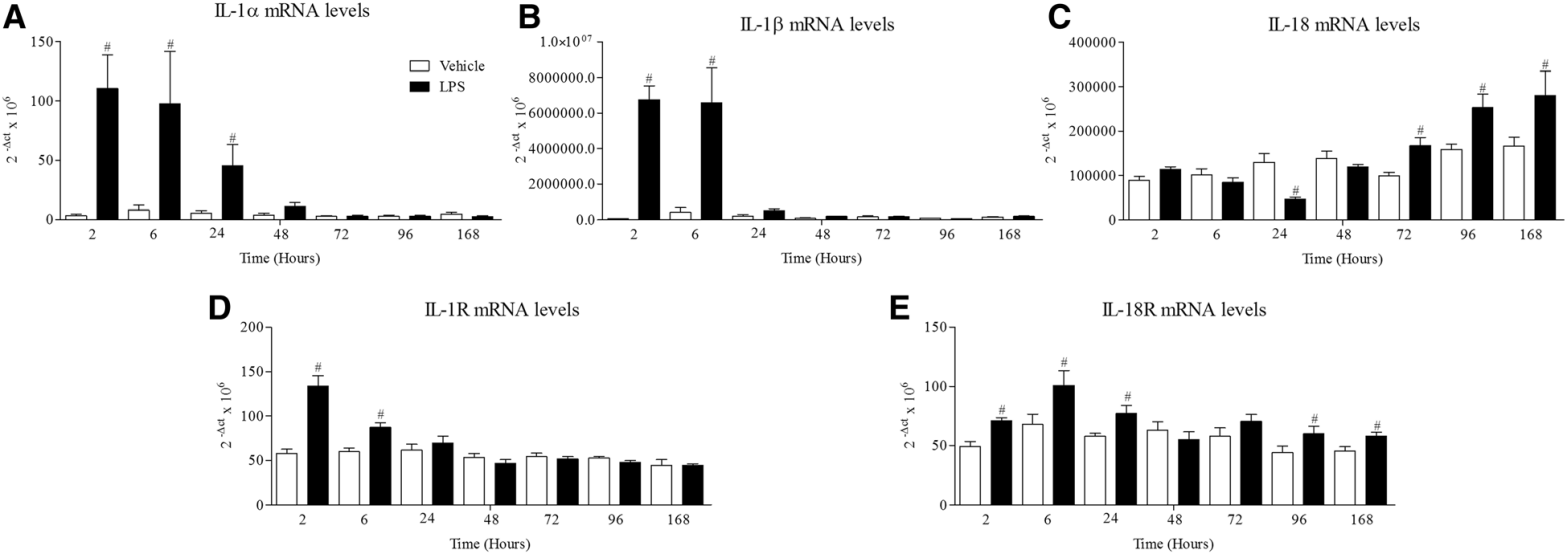
**Expression of IL-1 family (mRNA levels).** Male C57bl/6 mice were exposed to vehicle (aerosolised saline) or LPS (1 mg/ml) for 30 minutes. Lung tissue was collected at different time points after the exposure and mRNA expression of IL-1α **(Α)**, IL-1β **(Β)**, IL-18 **(C)**, IL-1R **(D)** and IL-18R **(E)** measured by real time PCR. Data are represented as mean ± S.E.M. for n = 6 animals in each group. Statistical significance was determined using Mann–Whitney U test. # = P < 0.05, denoting a significant difference between the LPS exposed and vehicle exposed wild-type groups.

**Figure 2:**
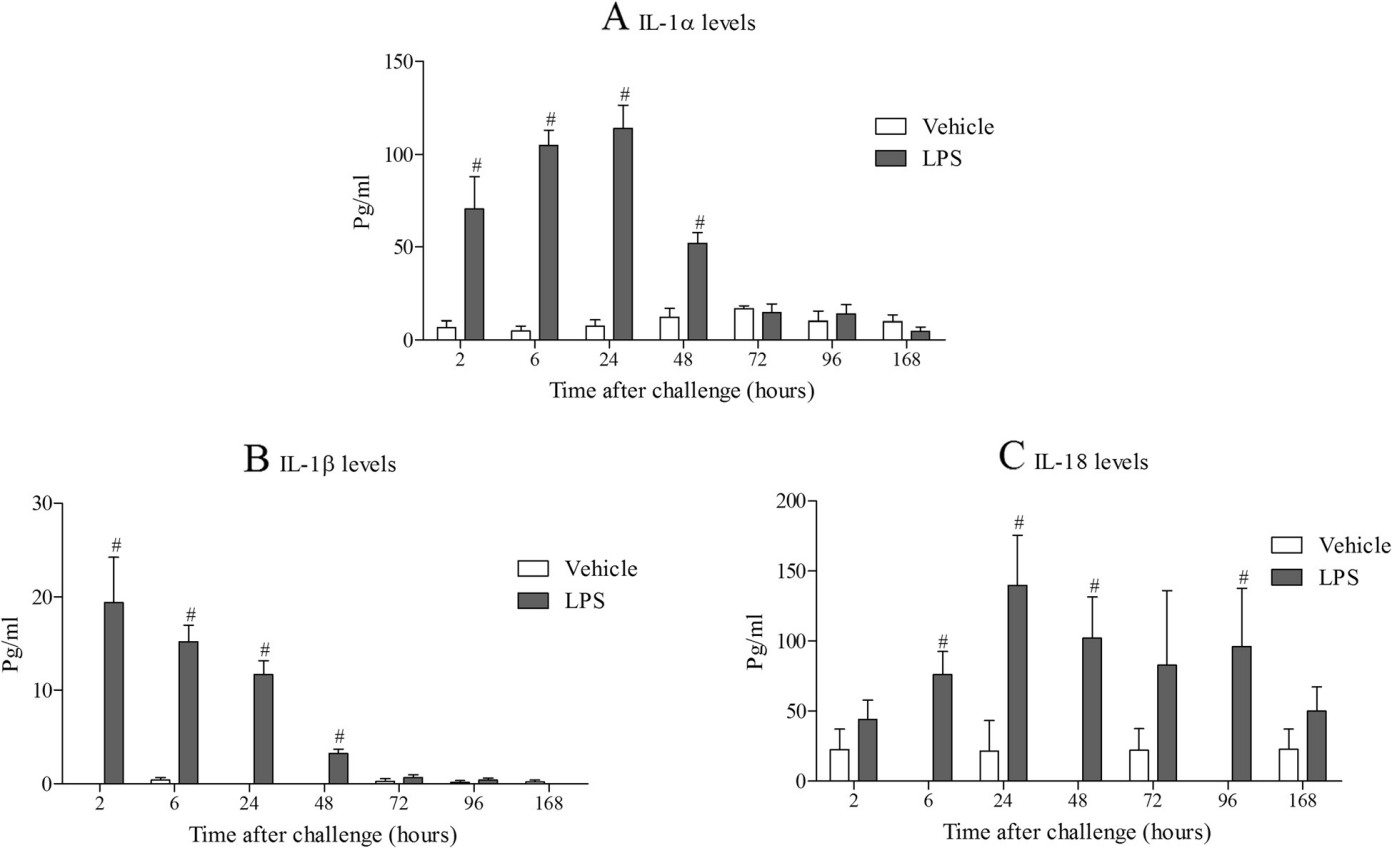
**Expression of IL-1 family (protein levels).** Male C57bl/6 mice were exposed to vehicle (aerosolised saline) or LPS (1 mg/ml) for 30 minutes. BALF was collected at different time points after the exposure and IL-1α **(A)**, IL-1β **(B)** and IL-18 **(C)** measured by ELISA. Data are represented as mean ± S.E.M. for n = 6 animals in each group. Statistical significance was determined using Mann–Whitney U test. # = P < 0.05, denoting a significant difference between the LPS exposed and vehicle exposed wild-type groups.

**Figure 3:**
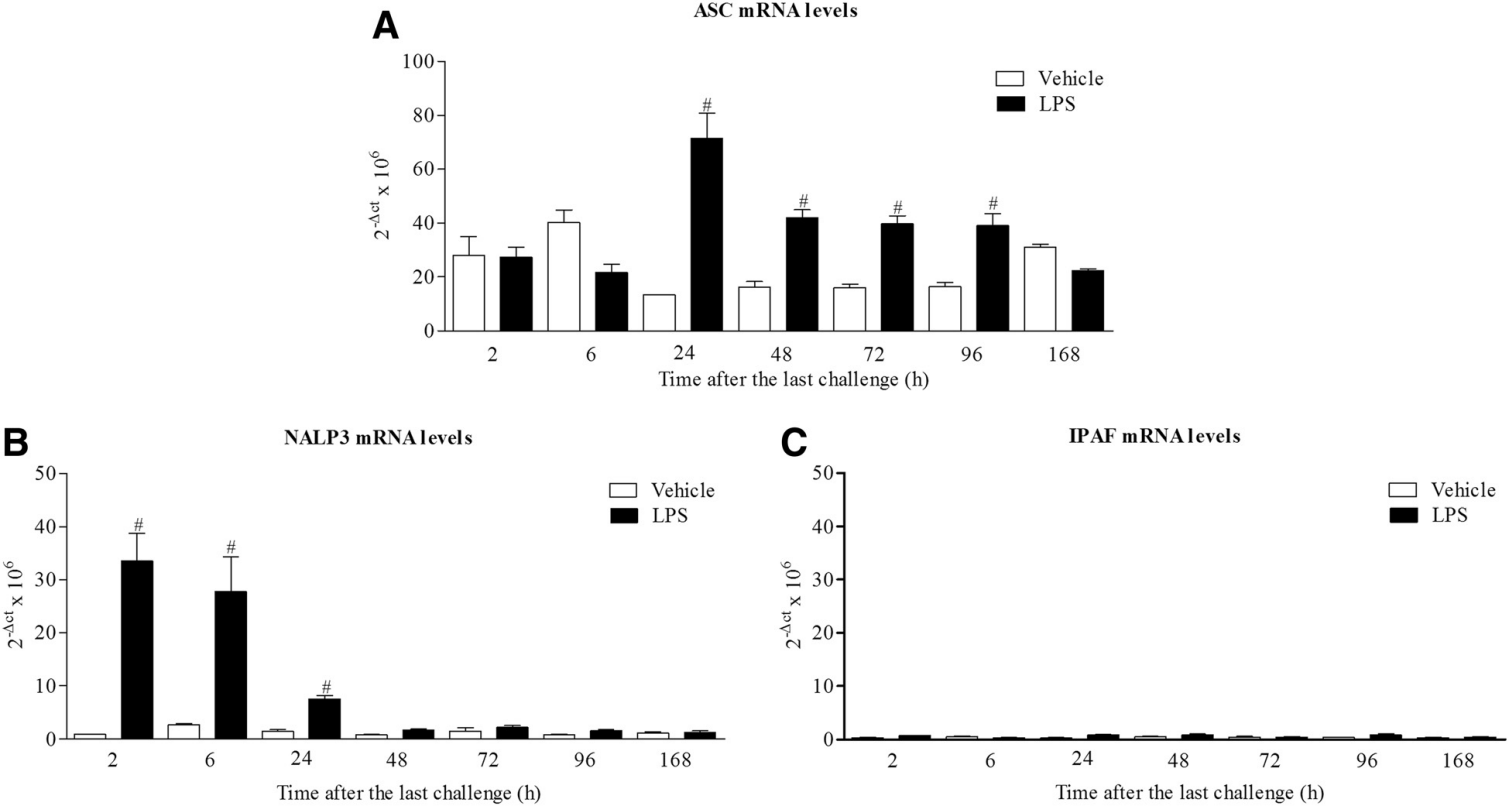
**Expression of ASC, NALP3 and IPAF mRNA levels.** Male C57bl/6 mice were exposed to vehicle (aerosolised saline) or LPS (1 mg/ml) for 30 minutes. Lung tissue was collected at different time points after the exposure. Lung tissue levels of ASC **(A)**, NALP3 **(B)** and IPAF **(C)** mRNA expression were detected by real time RT-PCR. Data are represented as mean ± S.E.M. for n = 6 animals in each group. Statistical significance was determined using Mann–Whitney U test. # = P < 0.05, denoting a significant difference between the LPS exposed and vehicle exposed wild-type groups.

**Figure 4:**
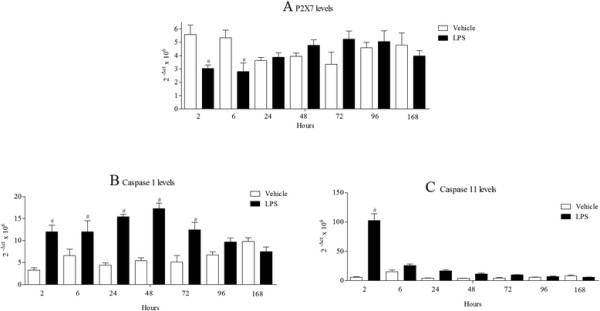
**Expression of P2X7, caspase 1 and caspase 11 mRNA levels.** Male C57bl/6 mice were exposed to vehicle (aerosolised saline) or LPS (1 mg/ml) for 30 minutes. Lung tissue and BALF was collected at different time after the exposure. Lung tissue levels of P2X_7_**(A)**, caspase 1 **(B)** and caspase 11 **(C)** mRNA expression were detected by real time PCR. Data are represented as mean ± S.E.M. for n = 6 animals in each group. Statistical significance was determined using Mann–Whitney U test. # = P < 0.05, denoting a significant difference between the LPS exposed and vehicle exposed wild-type groups.

Inhaled LPS caused a temporal increase in IL-1α, IL-1β, IL-1R and IL-18R, but surprisingly not IL-18, mRNA in mouse lung tissue (Figure 1). Indeed, it would appear at the 24 hour time point there was a decrease in expression of IL-18 mRNA. Measurement of BALF levels of the same cytokines revealed that all three cytokines are increased after LPS challenge compared to vehicle challenged, time matched control (Figure 2). Measurement of the machinery that is reported to be associated with the release of the IL-1 family of cytokines showed no change in IPAF mRNA, a transient decrease in P2X_7_ receptor mRNA, and increases in ASC, NALP3, caspase 1 and caspase 11 mRNA expression compared to lungs from time-matched, control treated mice (Figures 3 and 4).

### The role of TLR4 and Myd88 in LPS responses

**Figure 5:**
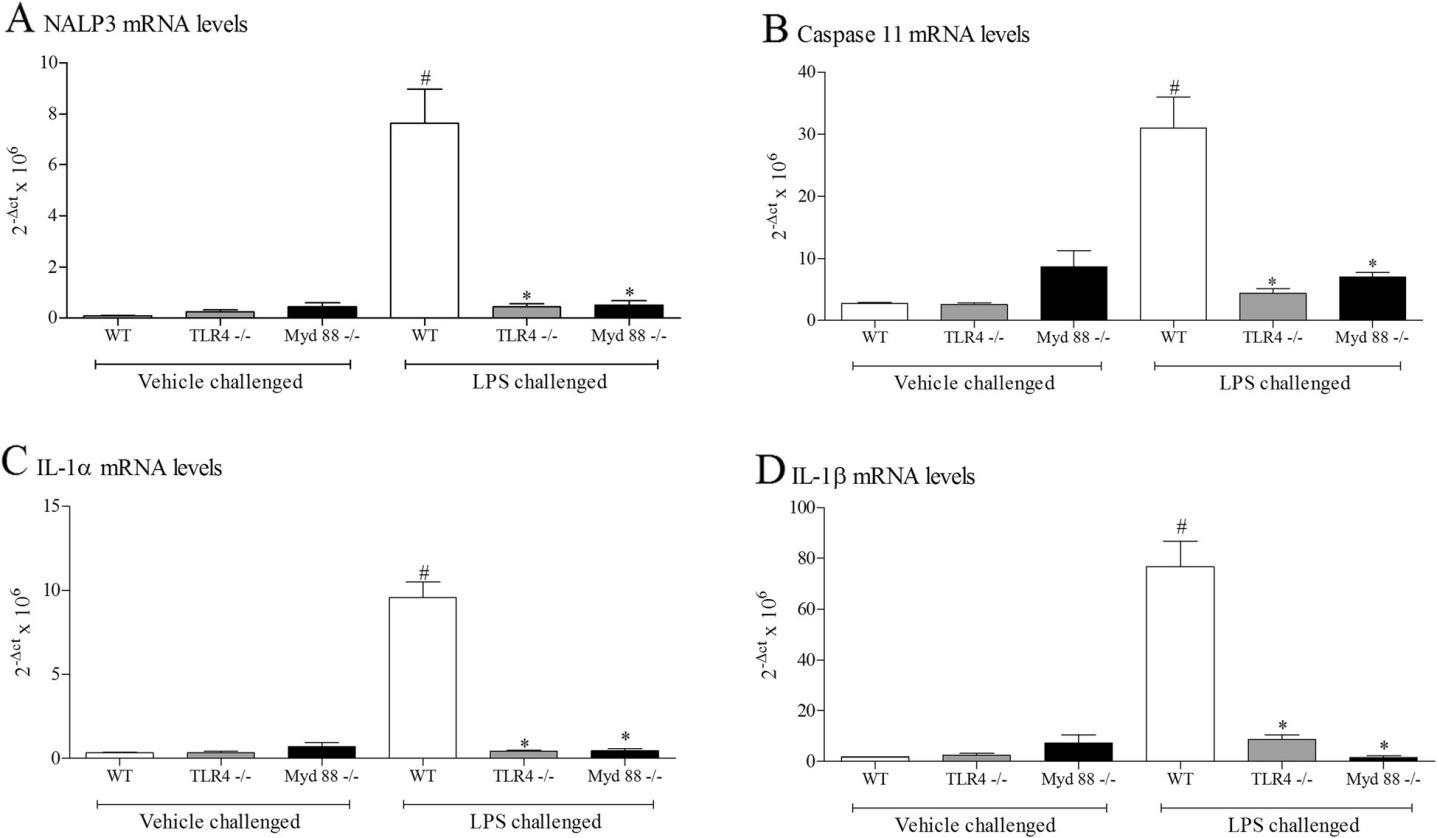
**Role of TLR4 and Myd88 in the expression of IL-1 family and associated markers.** Wild type, age matched control mice and GM mice (mice missing functional TLR4 or Myd88) were exposed to vehicle (aerosolised saline) or LPS (1 mg/ml) for 30 minutes. Lung tissue was collected 6 hours after the exposure. Lung tissue levels of mRNA expression were detected by RT-PCR NALP3 **(A)**, Caspase 11 **(B)**, IL-1α **(C)** and IL-1β **(D)**. Data are represented as mean ± S.E.M. for n = 4 animals in each group. Statistical significance was determined using Mann–Whitney U test. # = P < 0.05, denoting a significant difference between the LPS exposed and vehicle exposed wild-type groups; * = P < 0.05, denoting a significant difference between the LPS exposed GM and wild-type mice (one-way ANOVA).

Compared to wild type, age matched controls; the response to inhaled LPS was completely attenuated in mice missing functional TLR4 and Myd88 as expected. Levels of NALP3, caspase 11, IL-1α and IL-1β were increased 6 hours after LPS challenge in wild type mice and not in the KO mice indicating that the response observed is dependent on TLR4 and Myd88 (Figure 5). Parallel data was obtained when cytokine protein levels were assessed in the BALF (data not shown).

### Mechanism of cytokine release following LPS challenge

**Figure 6:**
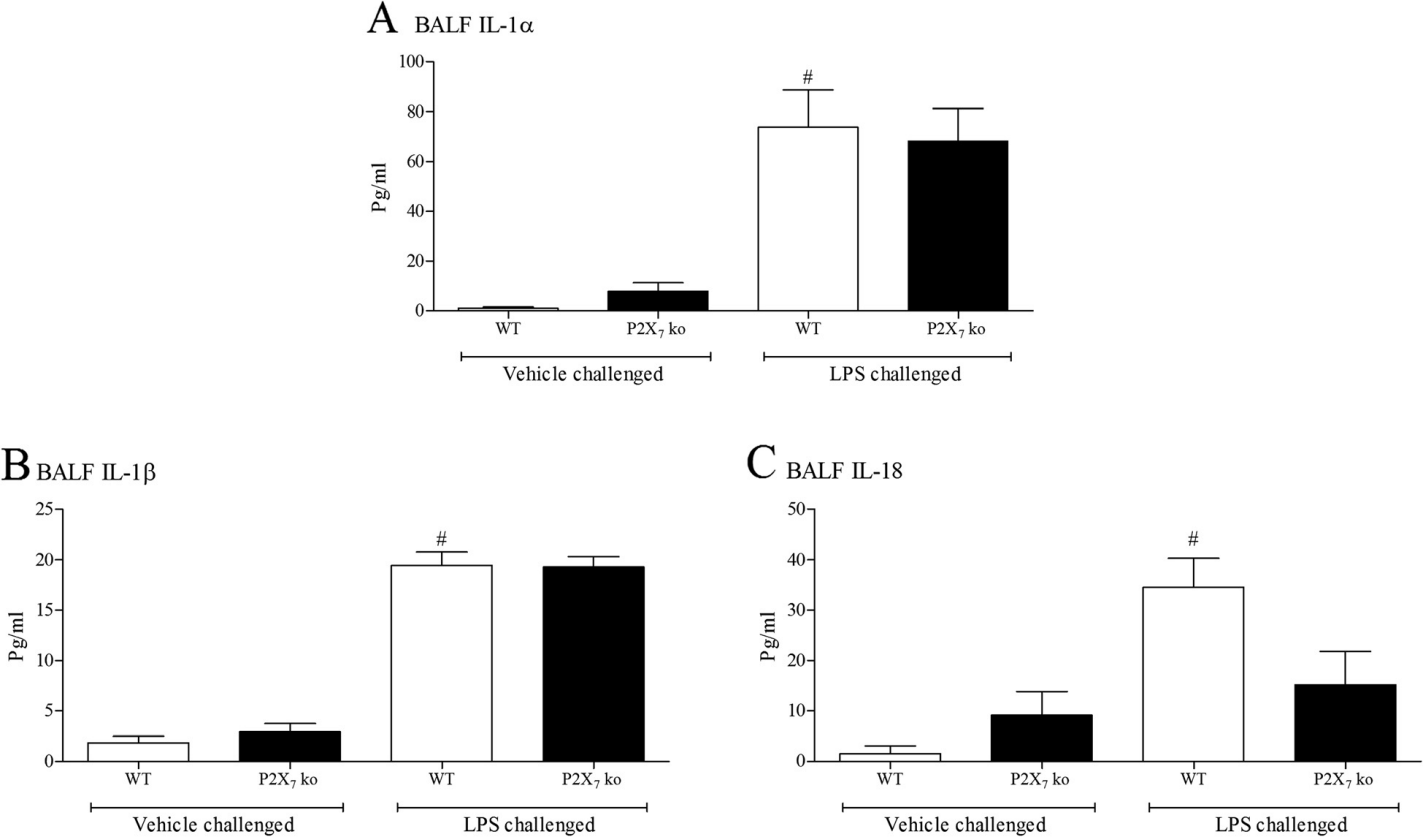
**Role of P2X**_**7**_**receptor in the expression of IL-1 family.** Wild type, age matched control mice and GM mice (mice missing functional P2X_7_) were exposed to vehicle (aerosolised saline) or LPS (1 mg/ml) for 30 minutes. BALF was collected 6 hours after the exposure and IL-1α **(A)**, IL-1β **(B)** and IL-18 **(C)** measured by ELISA. Data are represented as mean ± S.E.M. for n = 8 animals in each group. Statistical significance was determined using Mann–Whitney U test. # = P < 0.05, denoting a significant difference between the LPS exposed and vehicle exposed wild-type groups.

As discussed above, the P2X_7_ receptor has been linked to the release of IL-1 family cytokines in configured cell based assays. The LPS challenge caused a statistically significant increase in IL-1 cytokines in the wild type mice (Figure 6). The levels of IL-1α and IL-1β 6 hours after challenge were not altered in the P2X_7_ KO mice; the level of IL-18 did appear to be reduced, although this did not reach statistical significance (Figure 6).

**Figure 7:**
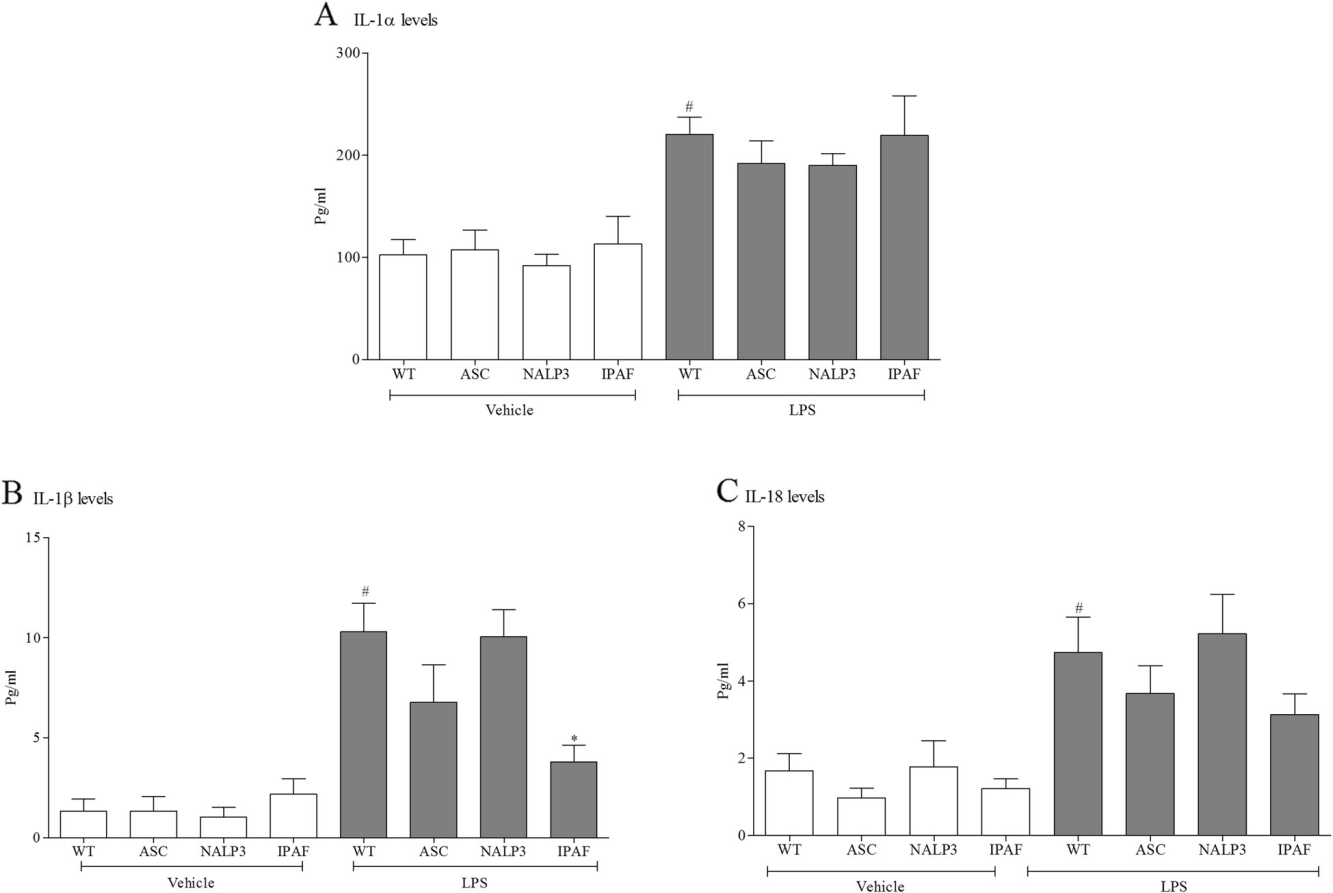
**Role of the inflammasome in the expression of IL-1 family.** Wild type, age matched control mice and GM mice (mice missing functional ASC, NALP3 and IPAF) were exposed to vehicle (aerosolised saline) or LPS (1 mg/ml) for 30 minutes. BALF was collected 6 hours after the exposure and IL-1α **(A)**, IL-1β **(B)** and IL-18 **(C)** measured by ELISA. Data are represented as mean ± S.E.M. for n = 8 animals in each group. Statistical significance was determined using Mann–Whitney U test. # = P < 0.05, denoting a significant difference between the LPS exposed and vehicle exposed wild-type groups; * = P < 0.05, denoting a significant difference between the LPS exposed GM and wild-type mice (one-way ANOVA).

The inflammasome proteins have been associated with processing and release of IL-1 family cytokines. Indeed results from the time course showed that mRNA levels of the NOD-like receptor NALP3 and adaptor molecule ASC were increased in the lung tissue after LPS challenge suggestive of a role in this process. However, data from a study in which we compared the levels of IL-1 cytokines in wild type mice to those measured in mouse lines missing key inflammasome proteins, showed that IL-1β and IL-18 levels were decreased in the IPAF and ASC KO mice but interestingly not the NALP3 KO mice (Figure 7). The levels of IL-1α were not altered in any of the GM lines tested (Figure 7).

As discussed above, dogma suggests that IPAF and ASC are involved in the maturation of IL-1 cytokines. To examine this we measured the levels of IL-1β in the lung tissue from the study with the inflammasome KO mice. As expected LPS challenge increased the mRNA level of IL-1β (4.4 to 64.4 2^^-dct^ × 10^6^), and this was not altered in the IPAF (69.5 2^^-dct^ × 10^6^) KO mice.

**Figure 8:**
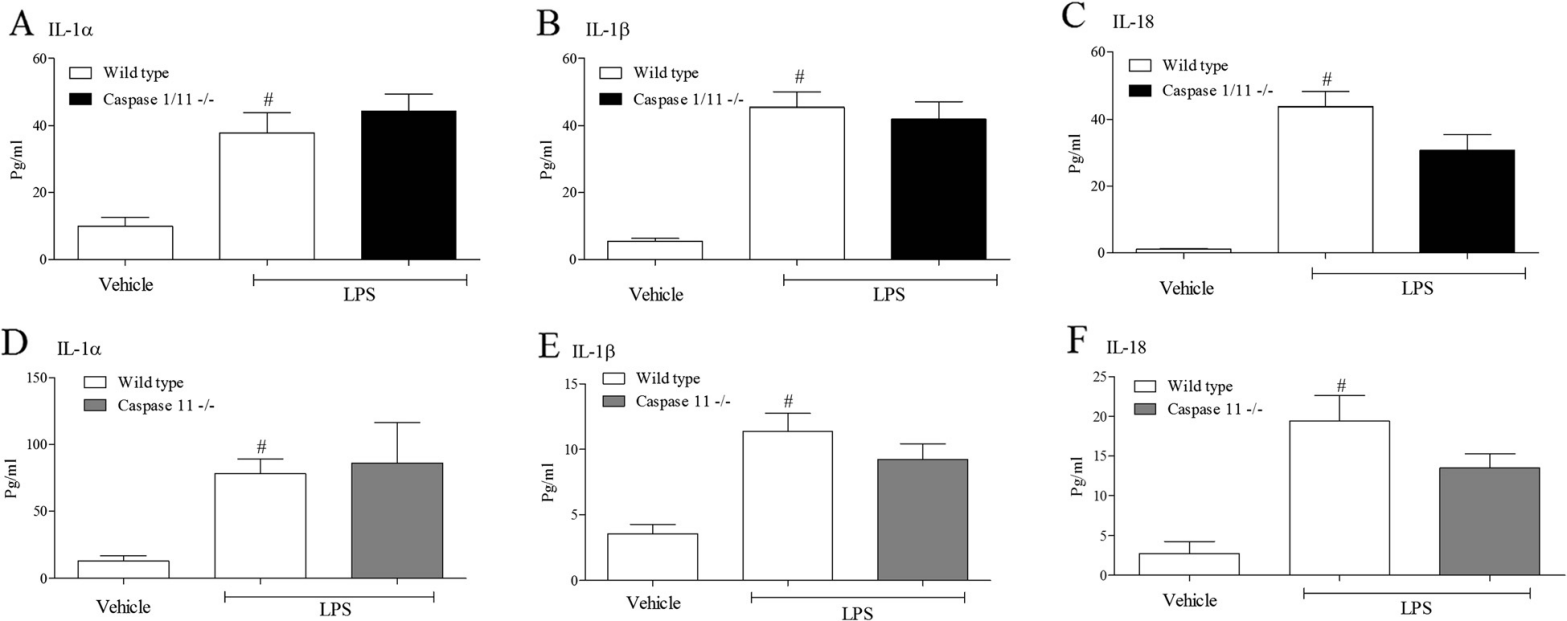
**Role of caspase 1 and 11 the expression of IL-1 family.** Wild type, age matched control mice and GM mice (mice missing functional caspase 1/11 and caspase 11) were exposed to vehicle (aerosolised saline) or LPS (1 mg/ml) for 30 minutes. BALF was collected 6 hours after the exposure and IL-1α **(A or D)**, IL-1β **(B or E)** and IL-18 **(C or F)** measured by ELISA. Data are represented as mean ± S.E.M. for n = 8 animals in each group. Statistical significance was determined using Mann–Whitney U test. # = P < 0.05, denoting a significant difference between the LPS exposed and vehicle exposed wild-type groups.

Caspase 1 and 11 have been reported to be central to the maturation of IL-1 cytokines and have been linked to IPAF, thus we wanted to explore the role of these enzymes in our system. In these studies, LPS challenge caused a significant increase in IL-1 cytokines in the wild type mice. In the caspase 1/11 and caspase 11 KO mice the levels of BALF IL-1α and IL-1β were not altered (Figure 8). In both GM lines the levels of IL-18 were reduced but this failed to reach statistical significance (Figure 8). There were no statistically significant differences in the cytokine levels of the vehicle challenged GM mice compared to wild type comparators (data not shown).

**Figure 9:**
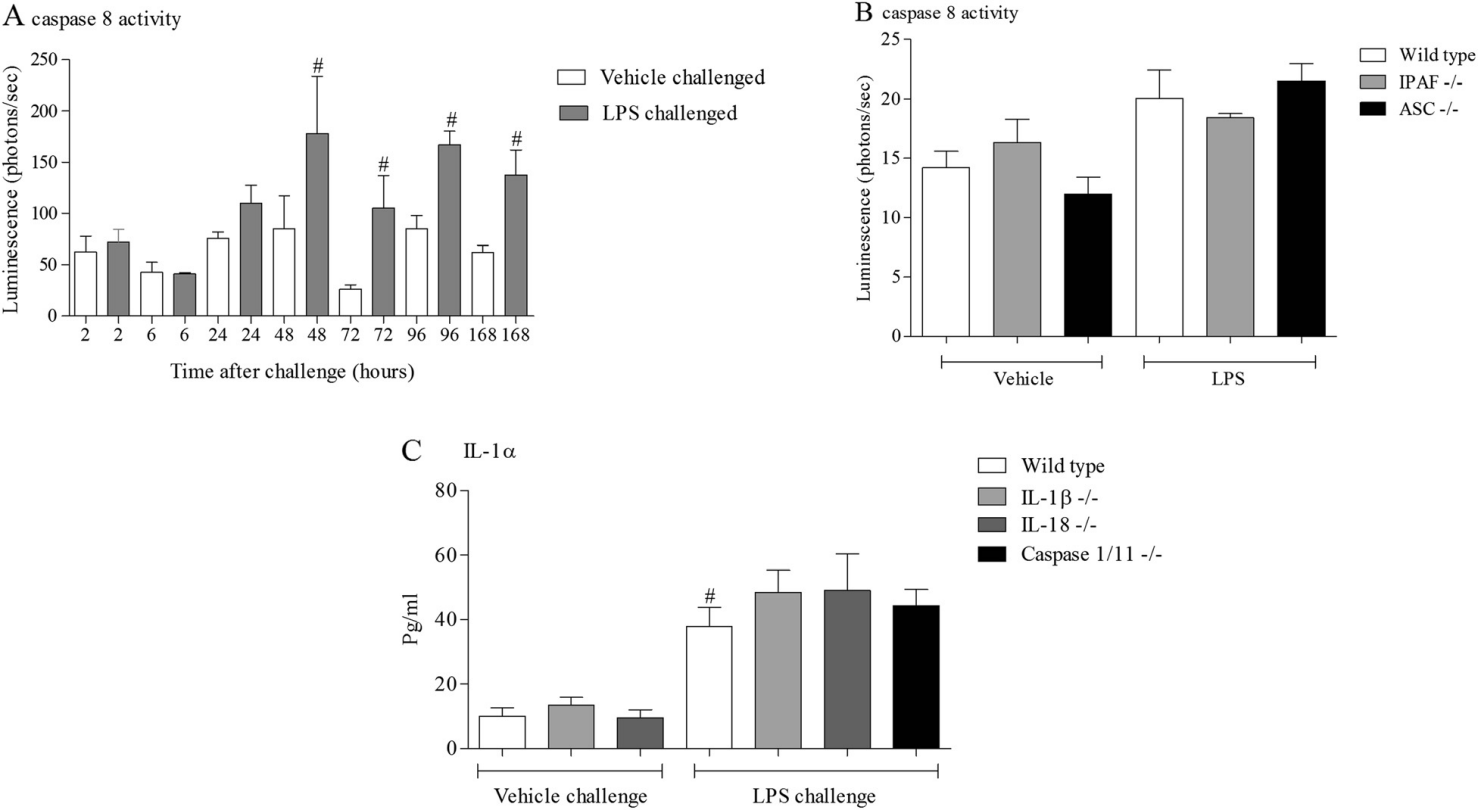
**Role of caspase 8 the expression of IL-1 family.** Male C57bl/6 mice were exposed to vehicle (aerosolised saline) or LPS (1 mg/ml) for 30 minutes. Lung tissue was collected at different time after the exposure and caspase 8 activity measured **(A)**. Data are represented as mean ± S.E.M. for n = 6 animals in each group. Wild type, age matched control mice and GM mice (mice missing functional ASC and IPAF) were exposed to vehicle (aerosolised saline) or LPS (1 mg/ml) for 30 minutes. Lung tissue was collected at different time after the exposure and caspase 8 activity measured **(B)**. Data are represented as mean ± S.E.M. for n = 6 animals in each group. Wild type, age matched control mice and GM mice (mice missing functional IL-1β, IL-18 and caspase 1/11) were exposed to vehicle (aerosolised saline) or LPS (1 mg/ml) for 30 minutes. BALF was collected 6 hours after the exposure and IL-1α **(C)** measured by ELISA. Data are represented as mean ± S.E.M. for n = 8 animals in each group. Statistical significance was determined using Mann–Whitney U test. # = P < 0.05, denoting a significant difference between the LPS exposed and vehicle exposed wild-type groups.

As it appeared that neither caspase 1 nor 11 were necessary for LPS induced IL-1 cytokines in the BALF, we studied the role of another caspase recently linked to the maturation and release of these cytokines, caspase 8 [30],[45],[46]. Caspase 8 KO mice are embryonically lethal [47] and there are currently no selective caspase 8 inhibitors suitable for *in vivo* studies. Thus we decided to measure caspase 8 activity to investigate its role in LPS induced BALF IL-1 cytokine levels. In the lung samples from the time course study we could detect an increase in caspase 8 activity (Figure 9). The temporal increase in activity did not appear to correlate with the presence of IL-1 cytokines in the BAL but appeared to be associated with the resolution of the cellular inflammation suggesting the increase is related to apoptosis of the white cells. Further evidence for the lack of a role for caspase 8 in the production of IL-1 cytokines is suggested by the fact that caspase 8 activity was reduced in the IPAF KO mice (Figure 9B).

IL-1β and IL-18 have been suggested to be linked to the release of IL-1α [48],[49]. To explore this in our model system we used IL-1β and IL-18 KO mice, with caspase 1/11 KOs as negative controls. As expected the LPS challenge caused a significant increase in BALF IL-1α which was, however, not altered in the KO mice (Figure 9C).

## Discussion

The IL-1 family of cytokines are known to play an important inflammatory role in many biological processes in the lung. Currently the mechanisms involved are unknown and understanding how these cytokines are produced is of paramount importance. Recently, research has focussed on the proposed 2 *step process* by which these cytokines are thought to be produced. In general the studies involve using configured cells grown from monocytes (i.e. blood) or harvested from body compartments (i.e. thioglycollate dosed peritoneal cavity, bone marrow) and when *in vivo* systems have been used often the end point used is death. However, it is well known that in the airway non-lethal, endotoxin challenge alone can cause the release of IL-1 cytokines in a range of species, including man [39]–[43]. Furthermore, LPS challenge models in man are becoming an increasingly popular investigative tool for new medications targeting COPD. Therefore, the aim of this study was to determine the mechanism by which the IL-1 family members are produced after activation of TLR4 by inhaled bacterial mimetic LPS.

In time course studies inhaled LPS triggered a rapid increase in IL-1α, IL-1β and IL-18 protein in the BALF but only increased IL-1α and IL-1β mRNA levels in the lung tissue. This would suggest that unlike IL-1α and β, IL-18 is not induced transcriptionally in this model system. Indeed, there is a strong signal for IL-18 mRNA in unchallenged mouse lungs and at the 24 hour time point following LPS challenge there appeared to be a decrease in IL-18 mRNA. This could because the mRNA is being used to replenish the normal stored levels of pro-IL-18, something that has been suggested in other lung inflammation models [50]. When we studied the expression of the machinery reported to be involved in the release of IL-1 cytokines, we found that ASC, NALP3, caspase 1 and caspase 11 were increased at the mRNA level, whereas P2X_7_ receptor expression was transiently reduced and IPAF levels were below reliable detection limits. Whilst, as far as we are aware, these markers have not been comprehensively measured in the mouse lung after LPS challenge, there are reports of similar increases in cultured cell systems [19],[51]. The decrease in P2X_7_ receptor mRNA expression is interesting. Previously a group has described an increase in P2X_7_ receptor protein expression in their mouse endotoxin challenge model, the difference could be down to the different experimental designs (i.e. dosing of LPS, timing of sampling) and measuring mRNA versus protein [52]. In addition, whilst we did observe a transient reduction in P2X_7_ receptor mRNA expression, this may not be indicative of changes in protein levels and other factors like levels of ligands and signalling/coupling status are more likely to be biologically important. On balance the expression data suggests that the endotoxin challenge is priming the machinery known to be associated with the release of IL-1 cytokine in the airway. One caveat, however, that could be argued is that these mRNA expression changes are due to an altered cellular burden observed after LPS challenge [39].

To determine if the increased expression was dependent on TLR4 and a key protein in the signalling cascade, Myd88, we repeated the experiment in GM mice missing functional proteins. The data clearly showed that TLR4 and Myd88 are required for the LPS induced increase in NALP3, caspase 11, IL-1α and IL-1β expression and whilst we cannot exclude the possibility that LPS enters the cell and directly interacts with proteins (similar to NODs), it seems likely that TLR4 and Myd88 play a crucial role in the response.

To begin to explore the mechanism by which IL-1 cytokines are released into the BAL, we explored the role of the P2X_7_ receptor. Although in these studies we challenged with LPS alone it is possible that this receptor is being stimulated by endogenously produced stimuli (i.e. ATP) and that this triggers the second signal known to cause the maturation of IL-1 cytokines. Indeed Monção-Ribeiro *et al.*[52] found that mice missing functional P2X_7_ had reduced levels of IL-1β. In these studies P2X_7_ KO mice had similar levels of IL-1α and IL-1 compared to age matched, wild type controls suggesting that endogenous P2X_7_ receptor activators are not produced in our model system. The reason for the discrepancy between data sets is not known but our data is in line with a previous finding that LPS challenge did not increase caspase 1 activity (a downstream marker of P2X_7_ receptor activation) [39]. Furthermore, we have detected an increase in BALF ATP levels in a smoke driven model [53], but no similar increase after LPS challenge (data not shown).

As discussed the IL-1 family of cytokines have been closely linked to inflammasome proteins; there are a range of publications linking the maturation and release of IL-1 cytokines to NALP3, ASC and IPAF. When we investigated their involvement in this system we found that although NALP3 expression was increased by LPS challenge, it was not necessary for the release of the IL-1 cytokines. IPAF, and to a lesser extent ASC, did appear to be required for IL-1β and IL-18, but interestingly not IL-1α. There are a number of reports that have, in general, have linked IPAF and ASC with mature IL-1 cytokine production in a caspase-dependent manner [54]–[57]. However, although we measured an increase in caspase 1, and its reported activator [44] caspase 11, surprisingly the caspase 1/11 or caspase 11 KO mice did not have reduced IL-1 cytokines. Whilst caspase 11 is not thought to process pro-IL-1β directly, it has been reported to cleave pro-caspase 1 into the mature active form and it has also been linked to the release of IL-1α [19],[20]. This data led us to the conclusion that the release of IL-1 cytokines was either dependent on another caspase or was independent of caspase enzyme activity. Recently it has been reported that caspase 8 can cleave pro-IL-1β [30]. We could not adopt our normal strategy and employ GM mice because blocking caspase 8 activity results in embryonic lethality [47] and as yet there are no selective pharmacological inhibitors with the correct profile for *in vivo* use. Thus we utilised the specific activity assay to look for an increase in caspase 8 activity and to determine if it was decreased in the IPAF KO mice (on the assumption that IPAF would be upstream of caspase 8). Whilst we did detect an increase in caspase 8 activity in the lung tissue, it appeared to temporally correlate with the resolution of the inflammation (i.e. associated with apoptosis) and any increase observed at the time points we measured IL-1 cytokines was not altered in the IPAF KOs. Together this data would suggest that the IL-1β and IL-18 measured in the BALF was dependent on IPAF but independent of caspase 1, 8 and 11 and would imply that other mechanisms are involved in the maturation of these cytokines as discussed previously. One candidate would be the matrix metalloproteinases [35] and we have previously reported that there is an increase in MMPs in the lung after LPS challenge [58]. However, data from a study in which we used a pan MMP inhibitor showed no change in IL-1β levels which would question this hypothesis [59].

Whilst we were able to determine that TLR4 and Myd88 were essential for LPS- induced IL-1α production in the BALF, we were unable to delineate further down the pathway. Reports have suggested that other IL-1 family members can act as chaperones for IL-1α but the data with IL-1β and IL-18 KO mice clearly showed that in this system this was not the case. Others have suggested that members of the calpain family of proteases are involved [60],[61] whereas recently Lukens et al. [62] have reported that RIP1 driven auto-inflammation targets IL-1α independently of the inflammasome. Further, investigation is required to determine the mechanism by which LPS induces an increase in BALF IL-1α after LPS.

**Figure 10:**
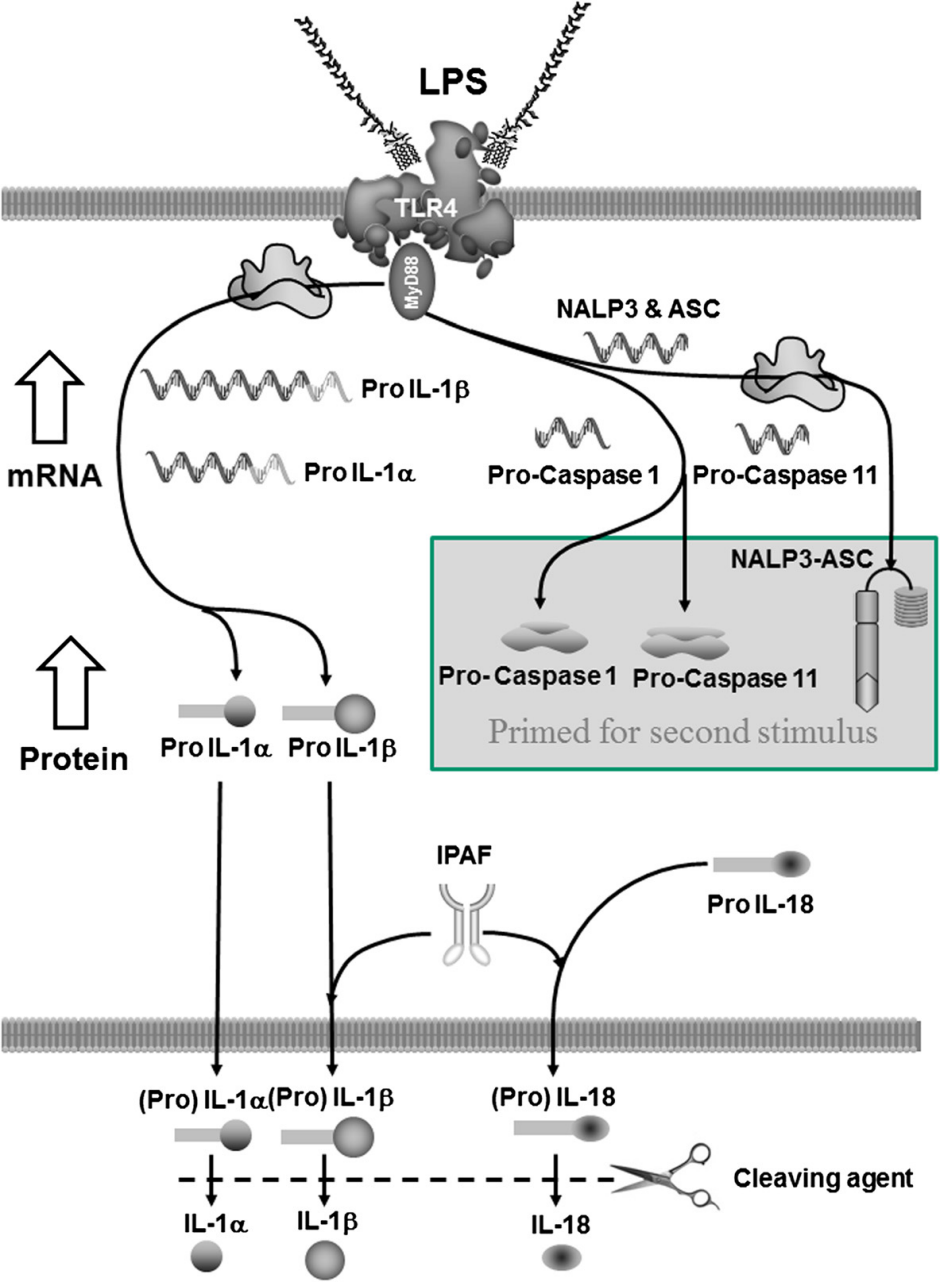
**Schematic representative of the*in vivo*data.** Our data suggests that in the mouse inhaled LPS activates TLR4 in the airway to trigger MyD88 dependent increase mRNA production of pro-IL-1α, pro-IL-1β, pro-Caspase 1, pro-caspase 11, ASC and NALP3, but not pro-IL-18 or IPAF. These increases in mRNA levels are likely to be associated with an increase in the corresponding protein levels. The pro-Caspase 1, pro-caspase 11, ASC and NALP3 are then primed ready for a second stimulus (i.e. via ATP-P2X7) and subsequent processing of pro-IL-1β and pro-IL-18. The LPS stimulus causes caspase 1/caspase 11 independent release of IL-1α, IL-1β and IL-18 into the airway lumen (either in the pro or mature form), of which the latter two appear to require IPAF. Pro-forms of the cytokines could be cleaved to the mature forms outside the cell.

In conclusion, the data suggests that activation of TLR4 increases levels of BALF IL-1β and IL-18 via an IPAF dependent and caspase 1/11/8 independent pathway. We have attempted to capture this in a schematic (Figure 10). Furthermore, it would appear that IL-1α is produced via a different mechanism. These are important, novel findings that further our understanding of this prominent family of inflammatory cytokines in the lung and their role in the innate immune system.

## Competing interest

The authors declare that they have no competing interests.

## Authors’ contribution

SE and MAB carried out the in vivo studies, LY-B, BD, SAM, EDD and VJ participated in the sample analysis. KAF generated the GM mice. SE, MGB and MAB conceived of the study, and participated in its design and coordination and helped to draft the manuscript. All authors read and approved the final manuscript.
